# Alteration of gene expression related to vulvar smooth muscle, extracellular matrix and innervation in vulvar lichen sclerosus: A pilot study

**DOI:** 10.1002/hsr2.208

**Published:** 2020-11-27

**Authors:** Qing Cong, Xiao Guo, Cenxi Liu, Fangfang Zhong, Jin Li, Long Sui

**Affiliations:** ^1^ Obstetrics and Gynecology Hospital, School of Life Sciences Fudan University Shanghai China; ^2^ Shanghai Key Laboratory of Female Reproductive Endocrine Related Diseases Shanghai China; ^3^ Shanghai Medical Center of Key Programs for Female Reproductive Diseases Shanghai China

## 1 INTRODUCTION

Lichen sclerosus (LS) is a chronic inflammatory disease with a potential for atrophy, destructive scarring, functional impairment, and increased risk of malignant evolution.^1^, ^2^ Notably, a significant number of LS patients are asymptomatic. It is most frequently seen in the anogenital area.^3^ Women with vulvar LS often present with severe pruritus and soreness of the vulvar and perianal areas.^4^ In advanced stages, there is destruction of the vulvar anatomy.^2^ If untreated, it is associated with a 2% to 6% lifetime risk of malignant squamous neoplasia of the vulva.^5^, ^6^, ^7^ Otherwise, potent topical corticosteroid is the gold standard for obtaining remission and reducing malignancy in vulvar LS.^7^, ^8^

Despite the possibility for treatment, the true etiology of LS remains unknown.^7^ In long‐standing and classic LS, the lymphocytic infiltrate is located under a band of homogenized collagen below the dermo‐epidermal junction.^9^ One study showed a significantly increase of pro‐inflammatory cytokines in LS patients.^10^

We hypothesize that the elevated inflammation of LS leads to alteration of smooth muscle, subcutaneous adipose tissue, extracellular matrix (ECM), and innervation of the vulva and aim to evaluate the changes of tissues by testing the expression of marker genes. To avoid the disturbance of various treatments, only untreated patients were included for this study.

## 2 MATERIALS AND METHODS

### 2.1 Reagents

Anti‐LMOD1 (A4585, ABclonal) and anti‐MYH11 (A4064, ABclonal) antibodies along with the other reagents, as described in the following paragraphs, were used.

### 2.2 Patient population

Current study was approved by the Medical Ethics Committee of Obstetrics and Gynecology Hospital of Fudan University (OGHFU No. 2018‐34). All enrolled patients signed a consent form.

Three women with typical lichen sclerosus by both clinical diagnosis and confirmatory biopsy were chosen. The detailed clinical information is described in Table S1. All the patients were untreated when the biopsy was performed.

Local anesthesia with lidocaine was applied in the most active sclerotic area and adjacent normal area in vulva. Disposable biopsy punches with the depth of 5 mm and diameter of 4 mm was used to biopsy. Samples were taken from either the most active sclerotic area or the adjacent normal area.

### 2.3 RNA isolation/quantitative RT‐PCR

TRIzol (Thermo Fisher) was used for total tissue and RNA isolation. Extracted RNA (500 ng) was converted into cDNA using the PrimeScript RT reagent Kit (Takara). Quantitative RT‐PCR (qRT‐PCR) was performed using an Applied Biosystems QuantStudio 5 and SYBR Green PCR Master Mix (Applied Biosystems). Fold change was determined by comparing target gene expression with the reference gene *36b4*.

### 2.4 Western blot

The samples were homogenized in 1% NP40 buffer with protease inhibitors. Lysates were then separated by SDS‐PAGE and transferred to Immobilon P membranes (Millipore). The membranes were blocked by 5% BSA in TBST and incubated with primary antibodies overnight and secondary antibodies for 1 hour. The membranes were incubated with ECL (Beyotime Biotechnology) and the bands were visualized by ChemiScope 6000 (CLINX). The intensities of the bands were quantified by ImageJ.

### 2.5 Statistics

The two‐way ANOVA test and the calculation of false discovery rate were performed by GraphPad Prism 8.0.1.

## 3 RESULTS

### 3.1 Remarkable changes of genes related to tissue damage in LS

**FIGURE 1:**
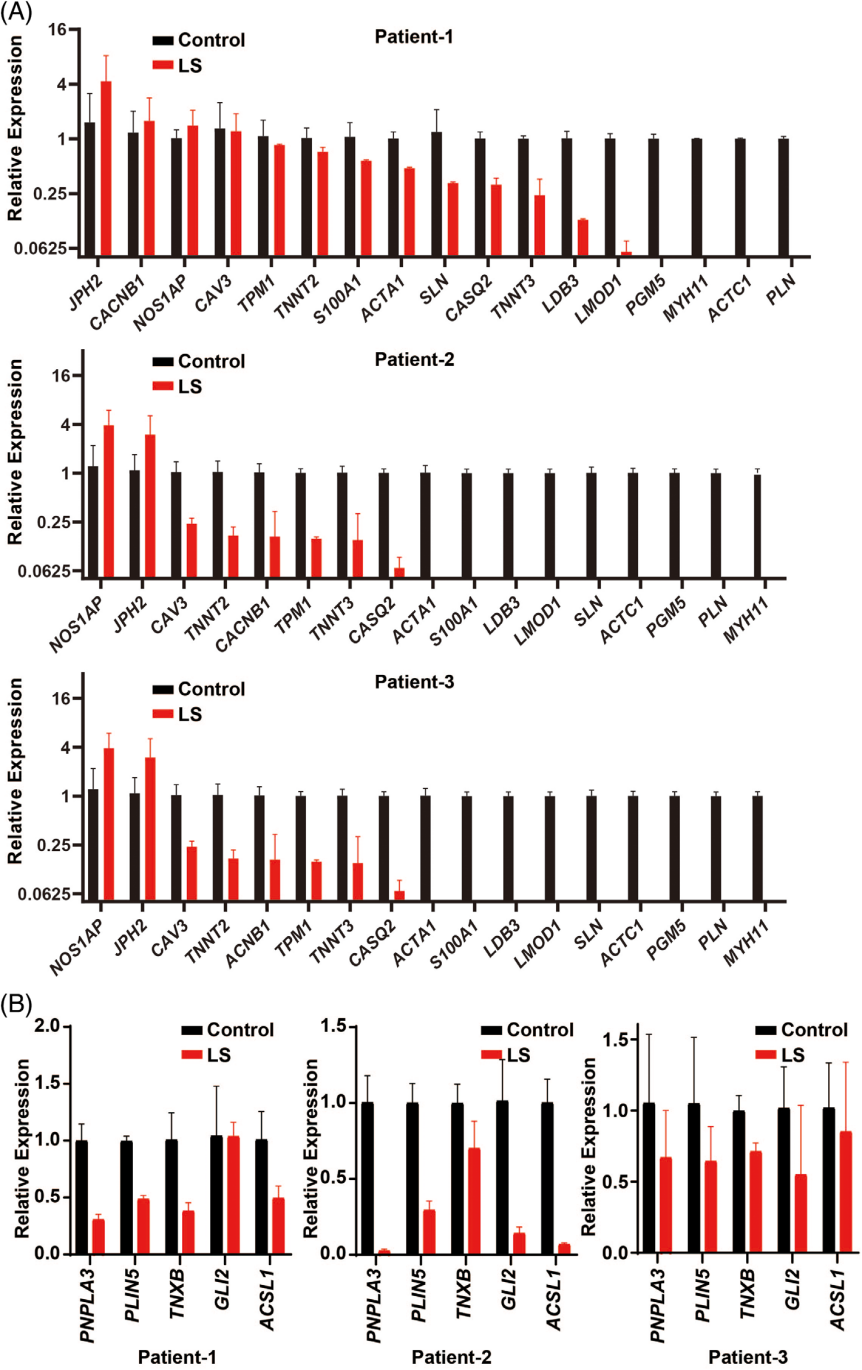
Decrease of genes related to subcutaneous tissue of LS. A, Downregulation of various genes related to smooth muscle in LS samples. B, Downregulation of genes related to adipose tissue, extracellular matrix, and nervous system

To explore the changes of smooth muscle in LS, we measured the level of mRNA in normal vs LS samples by qRT‐PCR. Among all the samples from the three patients, we identified a dramatic and consistent downregulation of genes related to smooth muscle structure or function (Figure 1A). We further investigated the changes of genes related to adipose tissue (*PNPLA3*, *PLIN5*, and *ACSL1*), ECM (*TNXB*) and the nervous system (*GLI2*). A similar trend was seen in genes related to these tissues, which were also downregulated in LS samples of all three patients (Figure 1B).

**FIGURE 2:**
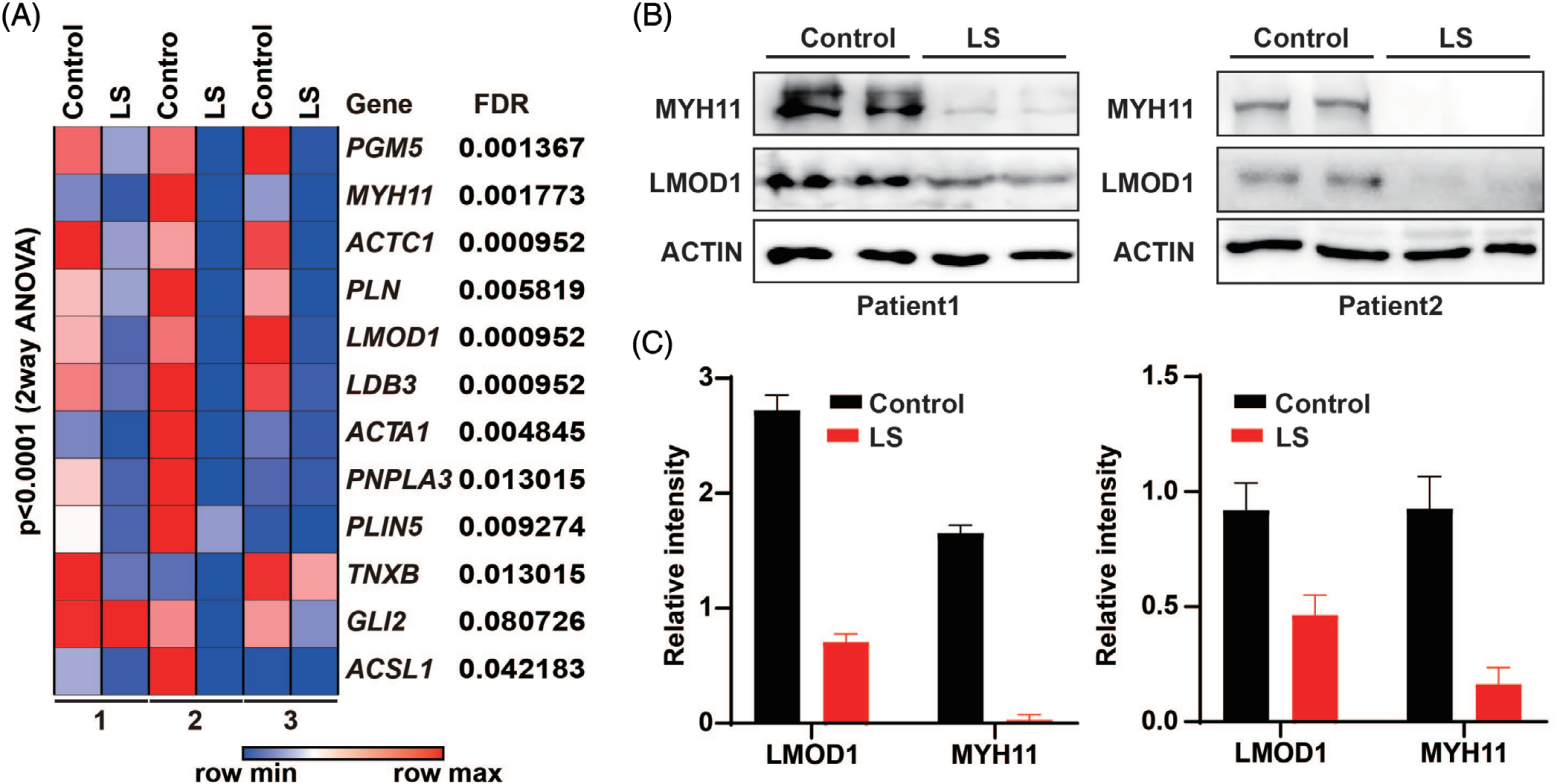
Significant downregulation of genes related to subcutaneous tissue of LS. A, Heat map showing changes of genes in three pairs of samples by color scale. False discovery rate (FDR) is listed on the right side of genes. The *P*‐value of two‐way ANOVA is indicated on the top of the heat map. B, Downregulation of MYH11 and LMOD1 in LS samples, shown by Western blot. ACTIN is the loading control. C, Relative intensity of bands in A, normalized to the intensity of ACTIN

To validate the significance of transcriptional changes among different patients, two‐way ANOVA test was performed and the false discovery rate (FDR) was calculated with pairwise normalization. A significant downregulation of genes related to subcutaneous tissue was observed in the LS samples, with FDR <5% for most of the genes (Figure 2A).

### 3.2 Downregulation of proteins related to smooth muscle in LS

As with the downregulation of mRNA levels for smooth muscle associated genes, we hypothesized that the protein products of these genes are also downregulated in LS. Due to the limited amount of proteins extracted from samples, we only managed to measure the changes of two representative proteins by Western blot. Based on the results of qRT‐PCR, we selected LMOD1 and MYH11 for the experiments. Similar to the changes of mRNA, both LMOD1 and MYH11 decreased in LS samples in comparison to the corresponding normal tissues (Figure 2B). The intensities of the bands were quantified and the difference between normal and LS tissue was identified among these two groups (Figure 2C).

## 4 DISCUSSION

Our study showed downregulation of gene and protein expression related to vulvar ECM, smooth muscle, and innervation. Up to 76.5%‐88.2% (13/17‐15/17) genes related to smooth muscle structure or function were significantly downregulated in three vulvar LS samples in comparison with normal skin from the same patients. Among them, *MYH11*, *LMOD1*, and their protein products were dramatically and consistently downregulated in LS samples, demonstrating that smooth muscle were damaged in LS.

*PNPLA3*, *PLIN5*, and *ACSL1* encode proteins important for lipid metabolism in adipose tissue. Changes in expression of these genes indicate subcutaneous adipose tissue damage in LS. ECM proteins are recognized as potential targets of immune response in LS^11^, ^12^ where, among them, tenascin has been reported in oral LS patients.^13^ We observed changes of *TNXB*, which encodes ECM protein tenascin XB, suggesting the damages of ECM in LS. *GLI2* encodes GLI family zinc finger 2, which is important for nervous development. Downregulation of GLI2 reveals the possible involvement of the nervous system in LS.

In summary, our study suggests the existence of smooth muscle, adipose tissue and ECM damages in vulvar LS. Small sample size makes it difficult to study whether the duration of the disease affects the gene expression. However, paired samples from the same patients as control were used to minimize the discrepancy of genotypes and to overcome the limitation of small sample size. RNA sequencing of vulvar LS can be performed in further study to systematically explore the etiology.

## FUNDING

This work was sponsored by Natural Science Foundation of Shanghai (20ZR1470900), Fudan University Zhuoxue Program, Ministry of Science and Technology of the People's Republic of China (2018YFA0801300) for study design, sample collection, and data analysis.

## CONFLICT OF INTEREST

The authors declare no conflicts of interest.

## AUTHOR CONTRIBUTIONS

Conceptualization: Long Sui, Jin Li, Qing Cong, Xiao Guo

Human tissue procurement: Long Sui, Qing Cong

Formal analysis: Jin Li, Xiao Guo, Cenxi Liu

Funding acquisition: Qing Cong, Long Sui, Jin Li

Investigation: Xiao Guo, Cenxi Liu

Supervision: Long Sui, Jin Li

Writing: Jin Li, Long Sui

 All authors have read and approved the final version of the manuscript.

 Long Sui and Jin Li had full access to all of the data in this study and takes complete responsibility for the integrity of the data and the accuracy of the data analysis.

## TRANSPARENCY STATEMENT

The corresponding authors, Long Sui and Jin Li, affirm that this manuscript is an honest, accurate, and transparent account of the study being reported; that no important aspects of the study have been omitted; and that any discrepancies from the study as planned have been explained.

## Supporting information


**Table S1**. Information of the patients.

## Data Availability

The data are available from Jin Li upon request.
