# Supplementary figures and images for: Alteration of gene expression related to vulvar smooth muscle, extracellular matrix and innervation in vulvar lichen sclerosus: A pilot study
Source: Health Sci Rep. 2020 Nov 27;3(4):e208. doi: 10.1002/hsr2.208 (PMC7695304; doi:10.1002/hsr2.208)

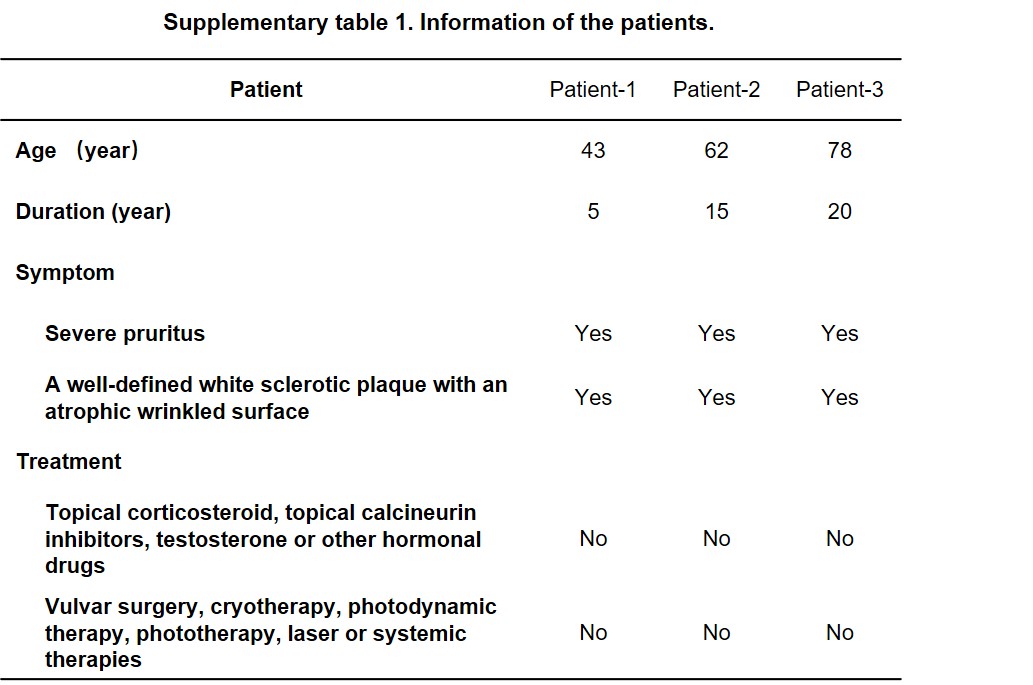

Supplement: Supplementary file 1 — Table S1. Information of the patients. [file HSR2-3-e208-s001.jpg]
